# Mass Carbon Monoxide Poisoning at a Daycare: A Public Health Lesson

**DOI:** 10.7759/cureus.66717

**Published:** 2024-08-12

**Authors:** Christopher Popiolek, Putt P Vithayaveroj, Chase L Jones, Natalie E Ebeling-Koning, John D DelBianco, Gillian A Beauchamp, Susan K Yaeger, Alexandra M Amaducci, Kenneth D Katz

**Affiliations:** 1 Department of Emergency and Hospital Medicine, Lehigh Valley Health Network/University of South Florida (USF) Morsani College of Medicine, Allentown, USA; 2 Department of Emergency and Hospital Medicine, Division of Medical Toxicology, Lehigh Valley Health Network/University of South Florida (USF) Morsani College of Medicine, Allentown, USA; 3 Department of Emergency and Hospital Medicine, Division of Pediatric Emergency Medicine, Lehigh Valley Health Network/University of South Florida (USF) Morsani College of Medicine, Allentown, USA

**Keywords:** unintentional poisoning, mass casualty incident, pediatric poisoning, carbon monoxide toxicity, carbon monoxide poisoning

## Abstract

Introduction: Carbon monoxide (CO) poisoning is a leading cause of preventable toxicity-related deaths in the United States. We describe a case series of 16 individuals who were exposed to CO due to a malfunctioning furnace at a Pennsylvania daycare, a state which did not mandate CO detectors in daycares.

Methods: An institutional review board-approved retrospective analysis was performed, and de-identified patient records were examined. Collected data included age, sex, race, ethnicity, CO concentrations, arrival time, time to hyperbaric oxygen center contact, and time to transfer and discharge.

Results: Emergency medical services transported 16 patients to a tertiary care emergency department (ED) with both adult and pediatric departments. Fourteen patients were 10 years of age or younger. Fifteen patients arrived within one hour. Sixty-two percent (N=10) were male, and 94% (N=15) identified as Hispanic. Emergency physicians, medical toxicologists, clinicians, interpreters, and volunteers from across the hospital system were mobilized to the ED to assist with management.

Conclusion: This large-scale daycare CO poisoning represents a potentially avoidable mass casualty incident among children and daycare staff and necessitated significant coordination of care. CO detectors in Pennsylvania daycares would provide early warning for staff, prevent or minimize toxicity, inform first responders, and better prepare EDs to handle similar situations.

## Introduction

Carbon monoxide (CO) is a colorless, odorless, tasteless gas which can only be detected with the aid of a CO monitor [1]. Unintentional CO poisoning remains one of the leading causes of preventable, toxicity-related deaths in the United States, and approximately 5,000 children present to the emergency department (ED) each year with CO poisoning [2,3]. In 2021, 28,900 people died worldwide from unintentional CO poisoning, with a higher incidence in males and in colder climates [4]. CO binds directly to hemoglobin in the blood with 250 times the affinity of oxygen [5]. This reduction in oxygen-carrying capacity and delivery, combined with interference of mitochondrial cytochrome oxidase, contributes to its clinical syndrome [6,7].

Initially, CO toxicity tends to present with nonspecific symptoms including dizziness, headaches, and nausea; however, more severe poisonings can progress to loss of consciousness and death [8]. The clinical severity does not directly correlate with blood carboxyhemoglobin (COHb) concentration, and, likewise, a decrease in COHb does not necessarily indicate an improvement in symptoms [9,10]. Long-term morbidity, including delayed neurologic sequelae (DNS) such as cognitive decline and movement disorders, may be caused by CO exposure [1]. Children are at great risk of CO poisoning as a result of their higher rate of metabolism and difficulty reporting symptoms, which may prevent early caregiver recognition and lead to misdiagnosis or delayed diagnosis [3]. In addition, the presence of fetal hemoglobin in neonates also leads to increased susceptibility to CO toxicity [3]. Standard management of CO toxicity includes the rapid removal of the patient from the source of CO, the use of 100% oxygen therapy via a non-rebreather face mask, and the consideration of hyperbaric oxygen (HBO) therapy in severe cases [11]. Despite the lack of randomized controlled trials for pediatric HBO therapy in CO poisoning, the current literature suggests that it is likely safe and may assist in averting DNS. The initial Glasgow Coma Scale (GCS) appears to be a predictor of DNS in cases of CO poisoning, although follow-up details were limited on many of the patients seen in the ED [12].

We describe a case series of 16 individuals who were exposed to CO due to a malfunctioning furnace at a Pennsylvania daycare, a state which did not mandate CO detectors in daycares at the time of this report.

This case series was presented, in part, at the American College of Medical Toxicology Annual Scientific Meeting on April 12, 2024.

## Materials and methods

Study design

This retrospective case series included 16 patients who were exposed to CO due to a malfunctioning furnace at a daycare and transferred to Lehigh Valley Health Network, Allentown, Pennsylvania, USA. This study was approved by the Institutional Review Board (IRB) of Lehigh Valley Health Network (approval number: STUDY00001312). The IRB determined that, as submitted, the project does not meet the regulatory requirements for human subject research but does meet the definition of research. Patient characteristics are shown in Table 1. Patients ranged in age from 1.8 to 54 years old, 14 of which were 10 years of age or younger, 62% (N=10) were male, and 94% (N=15) identified as Hispanic.

**Table 1:** Patient characteristics (N=16)

Characteristic	Total
Mean age (years)	4.8 (1.8-54)
Gender (% male)	62% (N=10)
Race	
Caucasian	6% (N=1)
Multi-racial	50% (N=8)
Other	44% (N=7)
Ethnicity	
Hispanic	94% (N=15)

Data collection

We performed an IRB-approved retrospective case series analysis. Patients, both adults and children, who were transferred by emergency medical services (EMS) to network EDs immediately following this mass casualty incident, as reported by EMS, were included in this analysis. Those who were not transferred to network EDs were excluded from the analysis. The de-identified data collected upon chart review included age, sex, race, ethnicity, CO concentrations, arrival time, time to HBO center contact, and time to transfer/discharge. Data was analyzed using basic descriptive statistics and was de-identified and stored in a password-protected spreadsheet on a secure drive within a secure folder.

## Results

EMS was called initially for a single pediatric patient with syncope. However, on arrival, additional symptomatic victims were identified. EMS CO detectors alarmed upon entry of the building, and subsequent fire department testing identified CO concentrations of 700-900 parts per million on scene. EMS transported 16 patients to a tertiary care center with both adult and pediatric EDs. Fifteen patients arrived within one hour. All but four patients were evaluated in the pediatric ED. Both an emergency physician and a consulting team of medical toxicologists managed the care of these patients; however, clinicians, interpreters, and volunteers from across the hospital system were mobilized to the ED to assist. The team members of the mass casualty incident assisted by sitting with the pediatric patients while awaiting parents, holding oxygen masks on children's faces, reading to children, and assisting with communication and care until parents arrived. Due to the volume of patients and the possibility of limited chamber access, several regional HBO centers were contacted to discuss five patients, all of whom were transferred for treatment (Table 2). The mean transfer time was 1.9 hours after phone acceptance to the HBO-capable facility. Criteria for consideration of HBO therapy included either a single COHb greater than 25%, signs and symptoms consistent with significant CO toxicity (loss of consciousness, syncope, or chest pain), consent by the patient or guardian, or acceptance at an HBO-capable facility. One patient was transferred to the HBO center due to a concentration of over 10% and a history of a congenital heart lesion. The mean COHb for the group of patients was 15.9%, with those transferred having a mean COHb of 22.0%. All patients not transferred were discharged on the day of ED presentation after several hours of oxygen therapy.

**Table 2:** Laboratory, encounter, and transfer data COHb: carboxyhemoglobin

Variable	Total
Mean COHb concentration (95% CI)	15.9% (12.3-19.5%)
Mean time from arrival to COHb result (hours)	0.98 (0.5-1.6)
Mean time from encounter to discharge (hours)	3.98 (0.85-5.25)
Mean interval between contact and hyperbaric center transfer (hours)	1.89 (0.48-4.9)
Hyperbaric center transfers	5

## Discussion

The challenges surrounding the coordination of care in mass CO poisoning have been previously described and were similarly demonstrated during this event [13]. CO concentrations obtained by EMS on scene were essential to guide ED care, particularly as the patients transported to the ED were largely children unaccompanied by parents. As with any mass casualty scenario in which multiple patients arrive in a short period of time, ED resources may become rapidly stretched beyond capacity.

At the time of writing, Pennsylvania does not require daycare facilities to have CO detectors. However, recent legislation enacted in 2016 requires CO detectors in other types of care facilities in the state, including nursing homes, personal residences, and assisted living residences (PA 2016 Act 48). As children represent a population that is vulnerable to CO poisoning, incidents such as the one presented here can be minimized or even prevented by the usage of CO detectors. The standard management recommended for CO toxicity involves removal from the source of CO exposure, administration of oxygen therapy, and consideration of HBO therapy, all of which rely on the early identification of toxic air concentrations of CO [11].

Mandated use of CO detectors in New York City increased home detection of hazardous CO concentrations sevenfold [1]. Despite this, data demonstrating that such detectors prevent morbidity or mortality are limited [6]. Christensen et al. demonstrated odds of CO poisoning were greater and medical outcomes were worse in both logistic and linear models among patients without CO detectors [14]. In a randomized controlled trial of ED interventions including education and the provision of a CO detector, those who received the intervention demonstrated "safe" CO detector use with a respiratory rate of 2.75 (95% CI: 2.1-3.7) [15]. While McKenzie et al. demonstrated the feasibility of prevention-focused education in the ED, an additional study emphasized the necessity of this education, as past surveys of patients in the ED report a lack of awareness of the significance of CO detectors, especially when compared to smoke detectors [16]. In the United States, only six states currently mandate the use of CO detectors in daycares [17]. Gordon et al. demonstrated that Wisconsinites aware of legislation regarding mandatory CO detectors were 2.82 times more likely to have a CO detector than those unaware [18]. In addition, none of the six states that mandate CO detectors in daycares currently is within the regions at highest risk due to colder climate, both the Northeast and Midwest [19].

This case series describing the management of a convenience sample of patients presenting to our hospital from a mass CO poisoning has limitations common to the study design. Our hospital did not assess all individuals exposed to CO on scene; thus, our data is limited to the 16 patients transported to our facility. Patients not assessed by our facility may have had different presenting features. Given our study evaluated a convenience sample of patients arriving from a particular incident, our findings cannot be extrapolated to all CO exposures. Like many hospitals, our institution does not have HBO therapy readily available outside of wound care centers, so the necessity of transfer of high-risk patients to an HBO-capable facility added complexity to the logistics of management which may not apply to other institutions. Access to a bedside medical toxicology consultation team was critical in this scenario, allowing for the rapid evaluation and disposition of the patients to complement the care provided by the ED clinicians. We note that many institutions would not have access to a bedside medical toxicology team to assist with the assessment, diagnosis, and management of a mass casualty toxicological exposure such as this, thus limiting extrapolation to other medical institutions. The cases reviewed in this case series were less severe compared to other case series reported in the literature; thus, our outcomes may not apply to more severe mass poisonings [20,21].

## Conclusions

This large-scale daycare CO poisoning represents a potentially avoidable mass casualty event that harmed children and staff and necessitated significant coordination of care. The consistent presence of simple, inexpensive CO detectors in locations with a density of vulnerable individuals such as daycare centers and schools would provide early warning for staff and potentially prevent or minimize toxicity. For incidents involving CO, first responders and EDs need to be prepared to handle mass poisoning scenarios. 
